# Investigating the effect of grit trait on performance and success in Hungarian athlete’s sample

**DOI:** 10.3389/fpsyg.2024.1283115

**Published:** 2024-04-12

**Authors:** Annamária Apró, Nikoletta Fejes, Szabolcs A. Bandi, Róbert Járai

**Affiliations:** ^1^Department of Psychiatry and Psychotherapy, Clinical Center, Medical School, University of Pécs, Pécs, Hungary; ^2^Center for basketball methodology and education, Pécs, Hungary; ^3^Doctoral School of the Institute of Psychology, Applied Psychology Programme, University of Pécs, Pécs, Hungary; ^4^Department of Cognitive and Evolutionary Psychology, University of Pécs, Pécs, Hungary

**Keywords:** sport psychology, adolescents, personality trait, perseverance, passion, long-term goals, grit

## Abstract

**Background:**

The aim of the present study is to translate the Grit questionnaire into Hungarian and validate specifically within the context of sports. The second goal is to assess the questionnaire in Hungarian as a pilot study in the athlete population and to compare the grit trait with the coaches’ athlete evaluation.

**Methods:**

Two hundred and sixty nine athletes, including 40 national team players, took part in the study, with an average age of 18.17 years (SD = 5.51). For the preliminary assessment, the Cloninger Temperament and Character Questionnaire (TCI-RH) was used; the coaches’ athlete evaluation was modeled on a talent map.

**Results:**

The confirmatory factor analysis confirmed the fit of the two-factor structure, and the internal reliability of the questionnaire scales also proved to be adequate. 2. There is no relationship between adolescents’ perceived grit and coach ratings. 3. The national team players achieved a higher grit score.

**Conclusion:**

Based on the psychometric indicators, the validity and reliability of the questionnaire proved to be adequate. Therefore, it is applicable and useful for psychological practitioners and researchers in the Hungarian population within the context of sports.

## 1 Introduction

One of the most fundamental and intriguing topics for professionals in the realm of any youth sports training system is finding the distinguishing psychological factors between prosperous and less successful players besides their physical capabilities - given that they undergo the same physical, tactical, and mental training - while identifying the players that possess the qualities to become professional athletes.

The study of the “grit” personality trait commenced in 2007 by Duckworth and colleagues, originating with the term’s definition, and the subject has now evolved to be a widely researched topic in psychology. Duckworth and associates (Duckworth et al., 2007) defined grit as a non-cognitive trait that influences an individual’s success and effectiveness through their committed perseverance toward long-term goals, sustained by passion even amid prolonged adversity. In essence, grit is described as “perseverance and passion for long-term goals” (Duckworth et al., 2007, 1087).

The emergence of this trait, however, is not confined to the 21st century: its origins trace back to the research questions formulated by William James in 1907 [as cited in Duckworth et al. (2007)]. James sought an explanation regarding individual differences that determine success and effectiveness; specifically, what personality traits contribute to certain individuals achieving more and performing better than their peers. Duckworth et al. (2007) reported that some professions are associated with certain personality traits. For instance, extroversion is considered to be indispensable in a sales position, while for a writer, it is irrelevant. This line of reasoning continued with success, asserting that specific traits were imperative for success and high performance - such as the grit trait among prominent leaders, regardless of their field of expertise (Duckworth et al., 2007; Duckworth and Quinn, 2009; Fejes and Járai, 2018; Birr et al., 2023).

Sturman and Zappala-Piemme (2017) define grit as maintaining a focused effort to succeed in a task, regardless of the challenges and the failure to overcome them. The grittier individuals are characterized by the fact that while others easily give up when experiencing setbacks, they remain in the “ring” and pull through in the face of adversity until they reach their goal (Duckworth et al., 2007; Fejes and Járai, 2018). By determination and perseverance, they are able to keep up with their initially more successful peers (Duckworth et al., 2007; Kelly et al., 2014). The observations also showed that being talented is not enough: determination, thorough work, and practice are also key requirements.

In their publication from 2007, Duckworth and colleagues stated that grittier individuals tend to stand up and build on setbacks and challenges while others are more likely to abandon their efforts when they fail. Their reluctance, determination, and perseverance allow them to stay on par with their initially more successful peers (Duckworth et al., 2007; Kelly et al., 2014; Birr et al., 2023). In a more recent publication from 2016, Duckworth articulates that the grit trait is similar to any other personality trait: it is biologically and genetically predetermined to a certain extent and also shaped by environmental influences. In terms of grit, perseveranceis 37%, and passion is 20% inheritable, however there is no gene specifically responsible for this trait. Nature and upbringing determine the emphasis of the grit trait, which may manifest in various areas of life (Duckworth, 2016).

A variety of recent researches has confirmed that in the academic field, grit can also be applied to predict academic performance [grade point average (GPA)^1^, Scholastic Aptitude Test (SAT)^2^ or American College Test (ACT)]^3^ (Duckworth et al., 2007; Duckworth and Quinn, 2009); and retention in the areas of military training, marriage, and sales (Eskreis-Winkler et al., 2014) as cited in Sturman and Zappala-Piemme (2017).

Strayhorn (2014) as cited in Sturman and Zappala-Piemme (2017) also found that grit prediction exceeds the predictive capacity of college and graduate school GPA and the SAT/ACT results - as it provides a more accurate prognosis (4% - see Duckworth et al., 2007) concerning the results and success of an individual.

To sum up, grit has been studied extensively in a variety of achievement domains and has been consistently linked to success and an array of adaptive correlates. Although, the Grit Scale (english version) was only used recently in educational context in a Hungarian sample (Csizér et al., 2023). The main goal of our study was to fill a gap and investigate the factorial structure and measurement in variance, as well as the psychometric properties of the Grit scale in the Hungarian language and sports context. We aimed to validate the use of the Grit scale as a tool for coaches and sport psychologists, given that grit appears to play a crucial role for coaches when making decisions about talent identification and development in athletes (Tedesqui and Young, 2020).

### 1.1 Expert performance and deliberate practice

Researchers in sports tend to attribute elite performance to genetic talent. However, they do not offer comprehensive genetic descriptions that would determine the causal processes involved in the activation and expression of dormant genes in developing athletes during exercise, leading to the appearance of characteristic physiological and anatomical features (innate talent). Ericsson (2006a, 2007, 2009a) claims that the evolution of the elite performance of healthy children can be calculated without the use of unique talent (genetic endowment) - except for the innate determinants of body size. However, the anthropometric differences are not negligible, the success of an individual in athletics is strongly determined by how much their anthropometric characteristics are similar to elite players’ athletic prowess. The determining role of physical properties varies by sport and it is important to note, for example, as encountered in basketball or handball, that one cannot limit athletic evaluation to only one anthropological model. It is imperative to establish separate models regarding different positions and genders (Rogulj et al., 2009). Significantly, fundamental differences about a player’s size and proportion are closely aligned to the various positions. In basketball, guards are typically the shortest team members, centers are the tallest in both genders, and forwards are usually in between the two heights (Ziv and Lidor, 2009).

Besides the anthropometric differences, Ericsson’s expert-performance approach shows that the distinctive characteristics of elite performers are adaptations to extended and intense practice activities that selectively activate dormant genes that all healthy children’s DNA contains. The expert-performance approach has provided accounts for elite performance in several domains of expertise, such as music, ballet, chess, and medicine (Ericsson et al., 1993).

Ericsson in his theoretical framework explains expert performance as the result of individuals’ prolonged efforts to improve performance while negotiating motivational and external constraints (Ericsson et al., 1993). In most domains of expertise, individuals begin their childhood with a regimen of effortful activities (deliberate practice) designed to optimize development. Even among elite performers, individual differences are closely related to the extent of deliberate practice. Research has found that engagement in deliberate practice is one of the best predictors of expert performance (Ericsson, 2006a). Deliberate practice refers to activities that require cognitive or physical effort, do not lead to immediate personal, social, or financial rewards, and are aimed at improving performance. Many characteristics once believed to reflect innate talent are the result of intense practice extended for a minimum of 10 years. During the first phase of learning, novices try to understand the activity (Fitts, 1967), cited in Ericsson et al. (2009a). Ericsson calls this stage everyday activities/everyday skills depending on the experience-performance (see more: Ericsson, 1998, 90). In the middle phase of learning, the learners start to eliminate large mistakes and they no longer need to concentrate as hard to perform the task. After a limited period of training and experience—frequently less than 50 h for most recreational activities—an acceptable standard of performance is attained. Without further modifications and improvements, this typically leads to a stable plateau of performance as the performance is automated. With special practice, performance speed improves significantly across various domains, leading to notable changes in both cognitive and psychological capacities.

Ericsson’s research shows that individuals who eventually reach expert levels of performance do not begin their training with an exceptional level of performance, nor do they suddenly attain extraordinary display of abilities at any stage of development (Bloom, 1985) cited by Ericsson et al. (2009a). The improvement of performance is gradual and generally takes several years of active pursuit to reach elite status. Even those who have historically been called “the most talented” usually do not win international competitions in less than a decade. Ericsson et al. (1993) stated that Simon and Chase’s (1973) “10-year rule” is supported by data from a wide range of domains: music (Sosniak, 1985), mathematics (Gustin, 1985), tennis (Monsaas, 1985), swimming (Kalinowski, 1985), and long-distance running (Wallingford, 1975). Children, adolescents, and adults who are committed to achieving expert-level performance begin working with coaches and suggest new exercises that continue to challenge them and provide feedback on performance and opportunities to repeat the task (deliberate practice). Deliberate practice requires a focus on improving performance and involves two types of learning: improving one’s existing skills and expanding the range and scope of one’s abilities (Ericsson, 2009b). According to another definition, “deliberate practice, which was defined as engagement in structured activities created specifically to improve performance in a domain” (Macnamara et al., 2014, p. 1608).

Gladwell (2008) in his viral book called “Outliers” introduces the “10,000 h rule” based on Ericsson’s theoretical work (Ericsson et al., 1993). Gladwell (2008, pp. 39–40) proposed that a minimum of practice hours was necessary and that this number was “the magic number for true expertise: 10, 000 h.” It has been treated as evidence that amateurs usually only practiced up to 3 h a week in their childhood. By the time they reached the age of 20, there were 2,000 h of practice behind them. Professionals gradually increased the number of hours per week up to 3 h a day or 20 h per week of exercise over a 10-year time interval. It takes the brain time to assimilate what is needed for masterful performance (Ericsson et al., 1993, 2006b; Gladwell, 2008). Although Ericsson et al’s. (1993) research showed that an extended period of training and practice was required to attain international-level performance, there was no evidence of a magical number. To win international piano competitions the first author estimated that around 25,000 h would be more accurate (Ericsson, 2013).

Ericsson’s expert-performance approach has found ample evidence that children and adolescents do not spontaneously engage in the deliberate practice that ultimately leads to maximal performance. Consequently, children need help to identify the appropriate training activities, to learn how to concentrate, and to find the optimal training environments. An early introduction to instruction and supervised training in the domain is associated with a greater likelihood of reaching the highest levels in many different types of domains (Ericsson et al., 1993). However, the maximization of deliberate practice is neither short-lived nor simple. The theoretical framework of expert performance explains the apparent emergence of early talent by identifying factors that influence starting ages for training and the accumulated engagement in sustained deliberate practice, such as motivation, parental support, and access to the best training environments and teachers. Ericsson et al. (1993) highlights three limitations: the resource constraint, the motivational constraint and the effort constraint. To maximize gains from long-term practice, individuals must avoid exhaustion and must limit practice to an amount from which they can completely recover on a daily or weekly basis (Ericsson et al., 1993; Baker and Young, 2014).

Duckworth examined Ericsson’s theory of practice (Ericsson and Pool, 2016) as cited in Duckworth (2016) and the grit trait, which concluded that grittier individuals not only learn more for Spelling Bee (Duckworth et al., 2011), the international spelling contest, but also have a higher GPA and tend to get better scores on standardized tests, such as the SAT or ACT. A noticeable difference in quality is also evident between contestants with varying placements (e.g.; Spelling Bee’s top three vs. the others). The positive outcomes might be influenced by the tendency for people with higher levels of grit to engage in higher amounts of deliberate practice (Duckworth et al., 2011).

According to the scoping review of Cormier et al. (2021), several recent studies explored the relationship between grit and deliberate practice. In four cases, whole grit scores were positively correlated with hours spent in deliberate practice (Larkin et al., 2015; Tedesqui and Young, 2017, 2018; Fawver et al., 2020), while CI alone was significantly associated with the number of miles run per week in long-distance runners (Cousins et al., 2020).

Achieving world-class performance without spending 10,000 h developing, practicing, and immersing oneself in the field of interest is difficult and uncommon. Most people can only amass 10,000 h if they enter a particular training system or program. Passion, practice, goal, and hope are the keywords of grit. To help an individual reach their goals, master their daily workout routine, to inspire and motivate them in a personalized way, they should create an effective talent program for youth training. Leaders must create an environment with excellent conditions, incorporate the know-how of experienced, professional teachers with cutting edge knowledge, and elaborate a training program of “hard and smart work.” As for the development of the player, this means that they not only need to work consistently for a significant amount of time, but the quality of the work has a substantial impact on further success.

Smart work is always intellectual; it improves our innovative and creative thinking skills and enhances productivity. These special training systems must work by the principles of international excellence standards, innovative approach, and original know-how in addition to applying evidence-based, practical experience, long-term approach, and winning philosophy to incubate talents. In the field of sports, such talent programs may require various knowledge areas of vast complexity: talent identification system, players’ performance assessment, training monitoring system, training program and periodization, recovery procedures, nutritional strategies, psychological care, injury prevention and rehabilitation system, the education of athletes about appropriate lifestyle and also the recruitment of performance people. In summary, the effectiveness of these systems depends on the individual approach to players according to their needs, the high volume of training (“smart and hard work”), the emphasis on training details, and the ability to build the “mindset of excellence,” which is possibly linked to the concept of grit. It involves persistence, consistency, and resilience, which are integrative concepts of perseverance known as empowering personality traits (Akbag and Ümmet, 2017). Grittier athletes are able to stay focused and also tend to respond positively to feedback from coaches and teammates. High levels of grit may help protect athletes from negative self-evaluations and when receiving ego-involving feedback, as their focus is on skill improvement and maintaining their passion and long-term quest for the sport (Moles et al., 2017).

### 1.2 Previous result related to grit in sports context

Research interest on this subject has significantly intensified over the past decade. Despite the indispensable findings of Duckworth and colleagues in 2007, our initial understanding was limited to the relationship between the Big Five personality traits and grit (a moderately strong, positive correlation with conscientiousness), while today we know that grit correlates moderately strongly with hardiness. The perseverance of effort (PE) factor of grit also shows a moderate relationship with the commitment component of hardiness (Kelly et al., 2014). Elumaro’s (2016) research revealed that grit was a predictor of sporting achievement while personality traits showed no significant differences.

Tedesqui and Young (2017, 2018) studied athletes’ level of perseverance (one of the dimensions of grit) and they found that it’s linked to the weekly quantity of deliberate practice, suggesting that individuals with high persistence cope better with the conditions of deliberate sports practice.

A study by Larkin et al. (2015) unveiled that among athletes participating in the Australian National Youth Soccer championship, those with a higher grit score showed greater sport-specific engagement (e.g., spending more time with training, racing, or in their free time, they have sports-related pastimes such as watching soccer matches or playing football in video games) in addition to having a better perceptual-cognitive performance (e.g., situational probability, pattern recognition, decision making).

Sellei’s (2018) personality psychological work also addressed grit. She concluded that the higher grit score correlates with low parental criticism (*r* = −0.333), concern (*r* = −0.209), and doubt (*r* = −0.315). In her cognitive psychological work, Sellei (2017) focused on the pattern of GRIT and VAL in athletes’ samples. The VAL value is a performance index used in basketball, which is described as “a player’s complex, full-match performance” (Sellei, 2017, 8). Although there was no statistically supported correlation between these two indicators, it was found that grit had a marginal significance in predicting national team membership.

Research indicates that higher levels of grit are associated with a number of psychological factors in sports. Martin et al. (2015) found a positive relationship between grit and engagement in sports. According to the studies, grit is associated with a reduction in burnout levels (DeCouto et al., 2019), perceived life stressors (Ford et al., 2017), maladaptive perfectionism (Fawver et al., 2020) and lower levels of sport-specific anxiety (Symonds et al., 2018). Experiences of pride are also associated with grit, but only when success is attributed to one’s own effort (Gilchrist et al., 2017). Crane et al. (2020) reported that lower levels of grit have been found to be associated with higher scores on general anxiety disorder measures. The grit subscales were also examined in sports context and the researchers found that CI and PE were both positively related to adaptive facets of sport-specific perfectionism, and inversely associated with maladaptive facets of sport-specific perfectionism (Dunn et al., 2021).

In the context of sports, Martin et al. (2015) related grit with commitment and staying in sports. From et al. (2020) reached a similar result as Fejes and Járai (2018). In their research examining athletes, they concluded that athletes achieved a higher grit score than non-athletes. From et al. (2020) focused on grit and conscientiousness in sports context because both traits have been connected to deliberate practice and performance (Barrick and Mount, 1991; Barrick et al., 2001; Ozer and Benet-Martínez, 2006; Duckworth et al., 2011; Eskreis-Winkler et al., 2014) and are highly related (Credé et al., 2017). They found that there was a high positive correlation between grit and conscientiousness and the elite athletes reported higher grit than the non-athletes control group (From et al., 2020). Reed and his colleagues (2012) investigated the relationship between grit, conscientiousness, and the transtheoretical model (TTM) of change for exercise behavior. The results showed that grit significantly predicted high-intensity and moderate-intensity exercise TTM stage while BFI Conscientiousness did not. The results suggest that grit is a potentially important differentiator of the TTM stage for moderate and high-intensity exercise (Reed et al., 2012).

According to the scoping review of Cormier et al. (2021), researchers examined differences in grit levels based on athlete sex and skill/competitive level. Several studies investigated the relationship between grit and sport performance and also examined relationships between grit and a variety of adaptive psychological constructs/characteristics and determinants of success in sport. It included relationships between grit and motivation, mindfulness, self-compassion, and deliberate practice. Sport researchers also assessed grit as it related to analogous personality characteristics including hardiness, resilience, mental toughness, self-control, and conscientiousness (Cormier et al., 2021).

González-Hernández et al. (2019) investigated the possible link between dark personality traits, and unhealthy behaviors and grit, notably the association between exercise addiction and grit in athletes’ sample. The results showed that the factor of Perseverance of Effort is positively correlated with addiction, and the other factor of grit, Consistency of Interests, did not present any kind of relationship. This seems to indicate that Perseverance of Effort is a trigger for addiction, while Consistency of Interests may help to self-regulate this behavior. In addition, younger athletes showed higher indicators of ambition to achieve their goals and a higher risk of exercise addiction, whereas gaining more experience with sports could facilitate the development of grit. In their other study (Nogueira et al., 2019), they strengthened this argument as the results showed that athlete men not only score higher for addiction levels but also narcissism (grandiosity feelings) and psychopathy (coldness) factors. They found that if signs of narcissism and Machiavellianism increase, perseverance of efforts grows too, and the likelihood of exercise addiction increases considerably. Consistency of interest appears as a protective factor, whereas Dark Traits of personality – especially Machiavellianism – constitute a risk factor.

Although grit has been consistently linked to adaptivity and healthy correlates, questions have been raised within the literature according to the conceptualization and measurement of grit. Credé et al. (2017) in their meta-analytic review of the grit literature found that the two dimensions that constitute grit, are the essential ingredients of success, assuming that perseverance of effort contributes to the achievement of excellence - despite failures and setbacks - and consistency in interests promote the commitment to deliberate practice in pursuit of the expert performance. According to their results, the higher order structure of grit was not confirmed, grit is only moderately correlated with performance and retention, and grit is very strongly correlated with conscientiousness. They found that the perseverance of effort facet has significantly stronger criterion validities than the consistency of interests facet and that perseverance of effort explains variance in academic performance even after controlling for conscientiousness. We have to mention that they also found inconsistencies in the assessment of the grit construct and they drew attention to the lack of confirmatory factor analyses to establish discriminant validity. They argue that the use of a general grit score might have limitations when it comes to predicting performance. Researchers have also found that the PE and CI subscales often have different relationships (and predictive power) with the same criterion variables – a finding that undermines the appropriateness of combining scores from the two subscales into a higher-order construct (e.g., Wolters and Hussain, 2015; Datu et al., 2017; Muenks et al., 2018).

Although one of the components of grit is perseverance, we have used it in a mainly positive context and we think there are circumstances in which it is maladaptive. (Datu and Fincham, 2021), cited in Birr et al. (2023) highlights that in circumstances where failure is inevitable, passion and persistence are not the right qualities to achieve success. Perseverance in the face of failures - whether repeated or unavoidable - can cause irreparable damage or loss. Therefore, blind persistence (Baumeister et al., 2003) cited in Birr et al. (2023) is not an optimal strategy when faced with tasks that cannot realistically be completed. This kind of persistence contradicts adaptability, since the individual is then unable to adapt his strategies to the situation, so it can show a negative correlation with consistency.

Taking the recent studies in sports context into account, grit was shown to be distinctive from other similar determinants of success such as hardiness, resilience, mental toughness, self-control, and in most of the studies, conscientiousness (Cormier et al., 2021). Conscientiousness, grit, and mindfulness strongly predicted resilience in sports context (Drury, 2019). Grit and self-regulation both share a positive relationship with resilience (Gupta and Sudhesh, 2019). It strengthens the argument that studies focusing on grit should continue and grit should be considered a useful and unique motivational tool by sport researchers.

### 1.3 Objectives and hypotheses

Several instruments are available to measure the grit personality trait, such as the 12-item Grit Scale (also known as Grit-O; see more Duckworth et al., 2007), the also 12-item Grit Scale for Children - GSCA (see more in Sturman and Zappala-Pierre, 2017) and the 8-item Grit-S (see Duckworth and Quinn, 2009). They were primarily available in English for measuring grit personality traits.

The main goal of our study was to examine the use of the grit scale in the sports context. We aimed to investigate the factorial structure and measurement invariance, as well as the psychometric properties of the Grit scale in the Hungarian language and sports context. We aimed to validate the use of the Grit scale as a tool for coaches and sports psychologists while we enhance the understanding of grit - as consistency of interests and perseverance of effort - and its role in sports performance and success.

The purpose of the present study is multi-faceted:

1. The adaptation of the original 12-item questionnaire into Hungarian as a pilot study for the sports population.
2. Comparing the athletes’ self-evaluation on the Grit Scale with the aspects of the coaches’ evaluation.
3. To verify the predictive power of grit regarding national team membership.

During the research, we assume that:

1. Grit Scale’s translated Hungarian version will show a more accurate fit with the original Grit Scale than the one used in our previous research (see Fejes and Járai, 2018).
2. There is a positive correlation between the evaluation by the coaches and the evaluation by the athletes.
3. The athletes of the national team achieve a higher grit score than their teammates, who are not members of the national team.

## 2 Materials and methods

### 2.1 Grit scale

Based on the theoretical conceptualization of grit, a brief and independent measurement scale was developed: The Grit Scale (Duckworth et al., 2007). The original Grit Scale, which measures individual differences in grit through self-report questionnaires, consisted of 12 items. The distinction between the two dimensions of grit [perseverance of effort (PE) and consistency of interests (CI)] is reflected in the subscales of the two primary self-report inventories used to measure grit: the Grit Scale (Duckworth et al., 2007) and the Short Grit Scale (Duckworth and Quinn, 2009) - both of which can be found on Angela Duckworth’s homepage^4^. The Grit Scale (English version) was only used recently in an educational context in a Hungarian sample (Csizér et al., 2023). The main goal of our study was to fill a gap by translating, validating, and examining the use of the Grit scale in the sports context. Due to scientific comparability and the larger international sample available, we took a decision to translate and validate the original Grit Scale (Duckworth et al., 2007).

### 2.2 Coaches’ athlete assessment

It should be considered that the grit personality trait did not have a significant relationship with the value of VAL – a performance index used and measured in basketball, which characterizes “a player’s complex performance throughout the game” (Sellei, 2017, p. 8). There was no statistically supported correlation between these two phenomena: *r* = 0.205 *p* = 0.199.

We tried to examine grit and effectiveness from another approach. Previously, Duckworth et al. (2011) measured the relationship between grit and performance among the participants of the Spelling Bee competition in the US. The result clearly indicated that the grit trait affects performance, leading to better placement. We used this method in our previous research (see Fejes and Járai, 2018) - so we compared the grit score of the athletes with their best ranking. However, we did not get the expected results because, in the case of sports, we cannot speak of an integrated qualification system (e.g., National Championship vs. Preparatory Competitions), so we chose to use the talent map written by the Basketball Academy’s specialists. In modern exercise and sports science, multiple methods are used to help experts identify talents and monitor their performance daily such as performance tests, motion analysis, collecting sensory data, etc. Coaches are also interested in statistics which analyze and rate the athletes as entities aligned with individual parameters. The talent map is a very simple method to summarize the test results and add the subjective assessment of an experienced coach who has been working with the player for a considerable time. “Our potential is one thing. What we do with it is quite another” (Duckworth, 2016, p. 14). The talent map describes the possible potential of the player. The talent map describes the possible potential of the player. It is necessary to add the subjective evaluation of the coach to the empirical data because, on the one hand, the unity of coach and athlete is essential and indispensable in sports: this includes the relationship and cooperation between them, as well as the meeting points, direction and goal of their motivation (Jowett and Ntoumanis, 2004; Kovács et al., 2021). On the other hand, acceleration as a phenomenon is common among youth athletes. In the case of young athletes, acceleration means faster-than-average growth and development. Because of the acceleration, they are taller, stronger, and more skilled than their peers. This can result in outlier performance, but only in that age group. However, the growth and development of their peers may catch up with them later, so the advantage caused by the acceleration disappears, and the sports results may lag (Balogh et al., 2015). Therefore the coach must be able to recognize and handle the accelerated condition, paying special attention to maintaining skills, motivation, and goals, and avoiding burnout. In the case of retarded or later-maturing athletes, the focus must be on maintaining motivation, providing development opportunities, maintaining self-confidence, and keeping them in the sport (e.g., if, compared to their teammates, the athlete is inferior in training or competitions, or in certain skills, the coach must know how to protect his athlete from premature dropping-out). According to this, grouping is based on the age group, gender, and level of talent. Players are divided among five categories, in which their coaches evaluate them and make predictions based on performance and ability. The most promising prospects are rated as players with international career opportunities, while the lowest-rated players are advised to change sports. Members of the national team receive the highest rating by default. Players are divided among five categories, in which their coaches evaluate them and make predictions based on performance and ability. The most promising prospects are rated as players with international career opportunities, while the lowest-rated players are advised to change sports. Members of the national team receive the highest rating by default. The map is created by the coach and the assistant coach in the pre-season period and supervised by the leader of the Academy. The map is dynamic and has to be discussed after the first half of the season (Rátgéber, 2017).

This method has been used at the Academy for several years.

The coaches were instructed to rate players on a scale from 1 to 5, based on their predicted potential:

1. A player with an international career opportunity
2. Potential to be a national team level player
3. 1st class Hungarian player
4. Amateur level player
5. Change the sport

“A player with an international career opportunity” is the highest rank and designates players who stand out from their age group in all indicators such as anthropometric characteristics, practice and match performance, motivation, skills and abilities, as well as mental strength. Youth national team players are automatically given the highest rating. The national team player category is the second, which shows that the given young athlete has the potential to be included in the national team as an adult professional player. The Hungarian first-class player is the third category, they are still outstanding athletes in their age group, who can reach the level of adult professional players by staying on a straight development curve. The third category includes those players who are expected to reach the plateau phase sooner and are capable of average performance in the given sport, which is not enough to become an elite player. The lowest classification is given to the players who are among the weakest both physically and mentally, for them it is recommended to choose another sport.

### 2.3 The questionnaire translation procedure

According to the protocols for translating questionnaires (Harkness et al., 2004), the translation procedure of the original English version of the Grit Scale to Hungarian, in our study, is the following:

1. The original 12 items of the Grit Scale were translated by two, independent bilingual translators, both of whom are experienced in sport psychology.
2. The two translated versions of the 12 Hungarian items were harmonized by a committee of three members with expertise in sports psychology.
3. The final items selected by the committee were translated back to the English language.
4. Small refinements were administered to a few items after the back-translation procedure.
5. The final versions of the Hungarian translation of the original 12 items were ready for validity and reliability testing.
6. The back-translation process was made by the original translators and the three harmonizers independently.

Minor adjustments were made in Hungarian language on all of the items based on the slight differences between the translators and harmonizers on the one hand, and considering the respondent feedbacks on the other.

### 2.4 Participants

**Figure 1:**
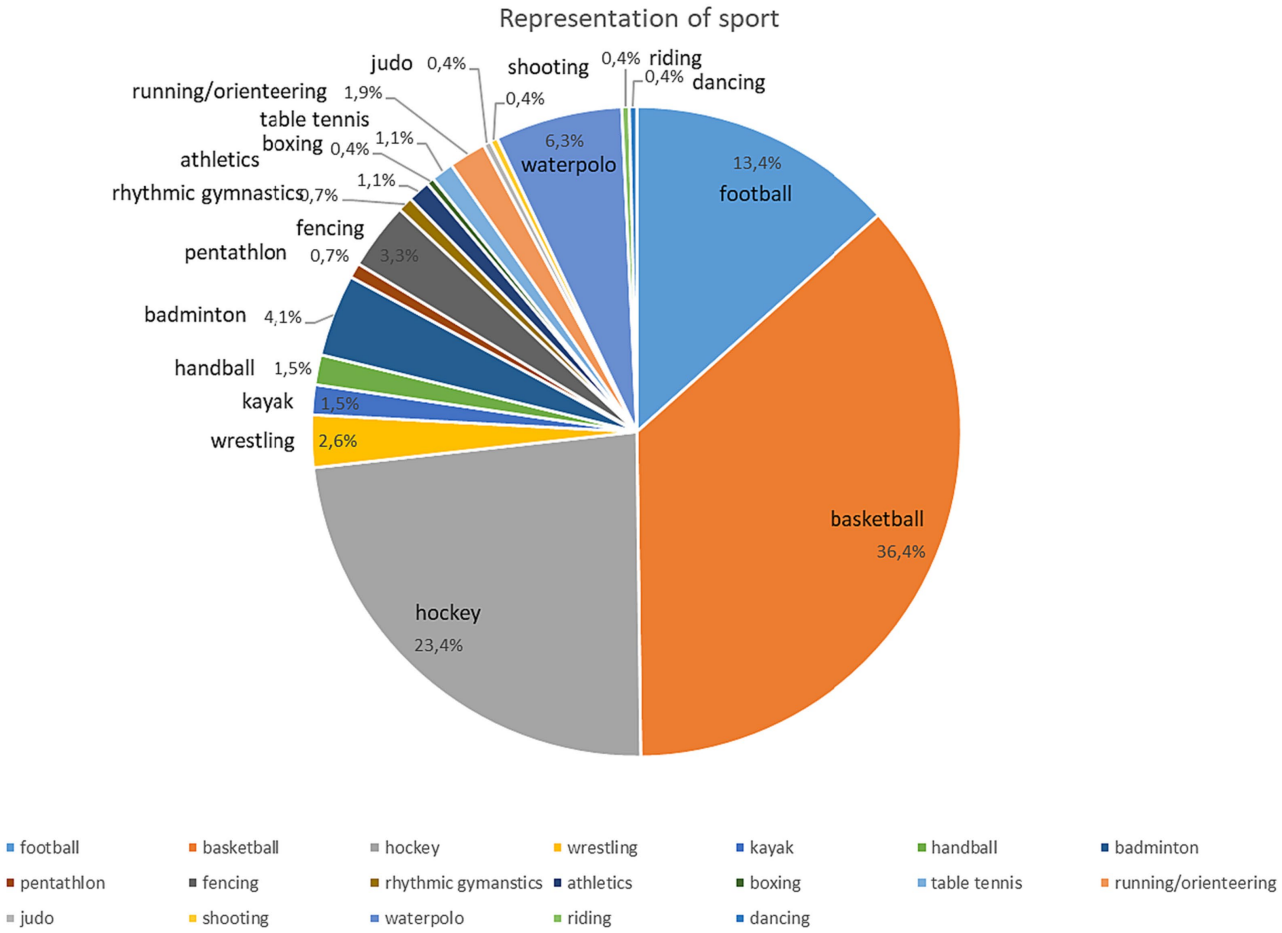
Representation of sports in the sample.

In our data collection, we gathered a database of 269 people. In the present cross-sectional study 269 athletes participated, with an average age of 18,17 years (the age range was 11–49 years; SD = 5.51), including 40 national team member athletes (average age of 23.07 years, SD = 4.39. Min.: 16 years max.: 38 years). 71.7% of the participants were male, 27.5% female, and 0.7% did not provide gender-related information. The majority of the roster was made up of primary or secondary school graduates. The athletes were participating in competitive sporting activities at an association/club; the national team member athletes - as the best of their age group - were individuals selected by the national federation of the sport and they were representing their country in international competitions, not a club. The sample included individual (e.g., badminton, pentathlon) and team sports (e.g., basketball, hockey, football) as well (see Figure 1).

### 2.5 Instruments

#### 2.5.1 Grit questionnaire

All respondents (*N* = 269) completed the Hungarian version of the Grit Scale (Duckworth et al., 2007) (see Supplementary Appendix 9A), which assesses the perseverance of effort (perseverance of effort; PE) and the consistency of interest (consistency of interest; CI) with 12 statements. Participants were required to rate each item on a 5-point Likert scale: 1 = I am not like this at all.; 5 = I am totally like this. A higher score suggests a higher level of grit. Cronbach-alpha value of original questionnaire: 0.85.

#### 2.5.2 Temperament and character inventory-55

In the shortened, 55-item Temperament and Character Inventory (TCI-55; Cloninger et al., 1993; Szabó et al., 2016), we used a dichotomous “true/false” response format (1 = true, 2 = not true). This personality questionnaire seeks to reveal the four temperaments and the three character factors (see Supplementary Appendix 9B). 113 athletes were subjected to the survey, of whom 29 were national team players, with an average age of 21.61 (SD = 6.19). 73.5% of the respondents were male, 23.1% female and 3.4% did not provide gender-related information. In terms of qualifications, nearly half of the participants had a high school diploma (43.6%), one-third of them were in elementary school (33.3%), and almost a quarter had a college or university degree (19.6%).

#### 2.5.3 Coaches’ athlete assessment

Coaches’ athlete assessment was based on a basketball academy’s selection system, which divided the players on a 5-point ordinal scale as follows: (1) player facing international career prospects; (2) player who has a possibility to become a national team member; (3) first-class Hungarian player; (4) amateur player; (5) sport change suggested.

Two coaches held this type of evaluation at a hockey association in Székesfehérvár.

#### 2.5.4 Demographic data sheet

We had the participants fill out a data sheet, asking for general demographic information: gender, age, the types of sports they participate in, the way of competing (individual vs. team-based), and we asked almost half of the examined sample about their sport ‘age’ (since when did you play sports), national team membership and education (typically for athletes aged 16 or older). The obtained results are characterized by the average sporting age (12.31 years) of the interviewed athletes (SD = 5.49 years; min.: 1 year max.: 44 years).

### 2.6 Procedure

All psychological research involving human subjects must be preceded by careful assessment of predictable risks and burdens to the individuals and groups involved compared to foreseeable benefits to them and other individuals or groups affected by the condition under investigation. We took every precaution to protect the privacy of research subjects and the confidentiality of their personal information. To this end, prior to the study, we requested and obtained permission from the Hungarian Ethics Committee (ref. nr. 2019–101).

In our research involving human subjects capable of giving informed consent, each potential subject was adequately informed of the aims, methods, and institutional affiliations of the researcher. The possible subject was informed of the right to refuse to participate in the study or to withdraw consent to participate at any time without reprisal.

During the study, athletes who were evaluated by the coaches and athletes participating in the test–retest process were given codes, as we strived to maintain anonymity. This means that we are unable and unwilling to identify participants. These persons received the questionnaire with a code name, and then we stored them in an envelope before processing the data. The key and the recorded questionnaires are stored locked away at the Institute of Psychology, and the data stored on the computer is protected with a code. Due to the confidential and personally sensitive nature of the information contained herein, this report is not to be provided to third parties. Most importantly, athletes under 18 could only participate in the research with parental consent, which parents could sign up for by signing the section next to their child’s name on a posted roster.

The procedure took place over several instances and locations/platforms. Athletes aged 18 and older completed the questionnaires online, in which the Grit Questionnaire (*N* = 108) and the TCI-RH (*N* = 83) were added to the demographic data sheet. 18% of these adult athletes participated in the test–retest procedure and filled out the questionnaire in person.

The participation of players in the 14–17 age group (*N* = 161) was based on a preliminary written information sheet and passive parental consent. This age group of individuals completed the test in person at an appointed time, in the halls of a given sports facility. The Grit Questionnaire and the demographic data sheet were also completed by this age group, and the coaches evaluated 60 players - but only 25 people were rated by 2 coaches. Other people (19% of adolescents’) filled out the TCI-RH questionnaire in paper-pencil form, and 15% of these adolescents participated in the test–retest process.

To test the reliability of the Grit Questionnaire translated into the Hungarian language, we used the test–retest reliability, in which the number of participants was 42—between the first and second test sessions, 3 weeks passed. The data was anonymized and participation in this study was voluntary - as mentioned above. 113 athletes were subjected to this part of the survey, with an average age of 21.61 (SD = 6.19). 73.5% of the respondents were male, 23.1% female, and 3.4% did not provide gender-related information. In terms of qualifications, nearly half of the participants were attending secondary school (43.6%), one-third of them were in elementary school (33.3%), and almost a quarter had a college or university degree (19.6%).

We handled missing data and questions that were not answered by removing the incomplete tests from the sample. No data was entered in these cases. A total of three athletes dropped out of the sample because of missing answers.

## 3 Data analysis

The data was analyzed with the statistical software IBM SPSS v.22 the open-source statistical software Jamovi v.1.6.16^5^, furthermore Microsoft Excel 2016. Factor structure was tested with the use of structural equation modeling (confirmatory factor analysis), with the use of the cut of values defined by Hu and Bentler (1998). Internal consistency was used to establish reliability with the use of Cronbach’s alpha. Test–retest validity was investigated with the use of Spearman correlations due to the non-normal distributions of the observed variables. The possible differentiating role of grit was assessed by binary logistic regression with several effect size measures (McFadden, Cox-Snell, Naglerkerkes, odds ratio) and with the use of the Mann–Whitney U test. The possible connection of grit with other related constructs, as a form of convergent validity was tested by both correlation analysis and linear regressions, both of them with multiple effect site measures, Criterion validity was also tested with correlational techniques.

## 4 Results

### 4.1 Factor structure of the Hungarian version of the Grit scale (Grit-HU)

**Figure 2:**
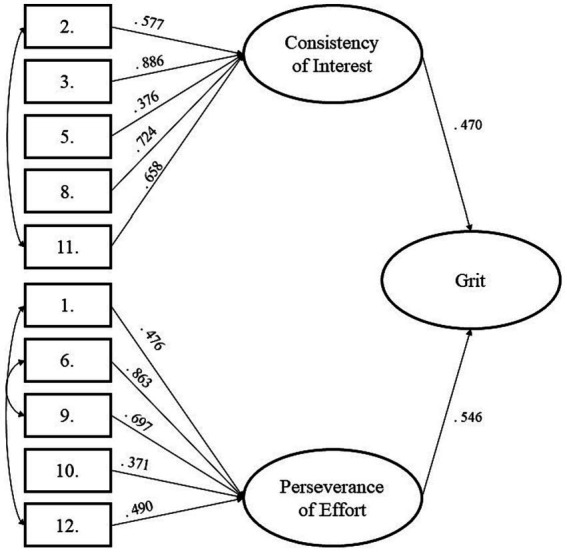
Confirmatory factor analysis of Grit-HU.

Confirmatory factor analysis (CFA) was applied to verify the proposed factor structure of the Grit Scale. In order to reconstruct the original design of the scale, we used a multilevel approach: two first-order factors (CI and PE) and one second-order factor (Grit) were included in our models. We proposed two alternatives: the first model (M1) included only the direct effects on the items to the first ordered factors and after the impact of CI and PE on the Grit. In contrast, in the second model (M2), three residual covariance relationships were applied between the items (see Figure 2). Based on Hu and Bentler (1998), according to the analysis results, M2 yielded satisfactory fit indices (see Supplementary Appendix Table A1), therefore, we accepted it as our final model. The Grit Scale’s Hungarian factor structure proved to be primarily similar to the original version with two significant changes: the fourth item (“Setbacks do not discourage me.”) and the seventh item (“I often set a goal but later choose to pursue a different one.”) showed no significant loading to any of the factors. Consequently, we chose not to involve them in evaluating the factors and the total score of the Grit Scale. These changes also indicate that in the following analyses, we used the means of the items of the factors and the full scale to ensure the extensive comparability of our results. To summarize our findings, the applied CFA provided a similar factor structure in the case of the Hungarian version of the Grit Scale (Grit-HU) with some relevant modifications.

### 4.2 Reliability of the Grit-HU

Two approaches of reliability analysis were applied to verify the structural and temporal stability of the scale. First, we used an internal consistency analysis employing the estimation of Cronbach’s alpha values. The internal consistency was found to be questionable in the case of PE (*ɑ* = 0.682; CI: 0.612–0.734), and acceptable concerning both the CI (*ɑ* = 0.768; CI: 0.721–0.808) and the whole scale (*ɑ* = 0.741; CI: 0.710–0.792). Based on Fornell and Larcker (1981) and Kline (2000) values above 0.7 indicate good reliability, the Hungarian version of the Grit Scale’s (Grit-HU) internal consistency is most acceptable for assessing grit and its components.

As another approach to testing reliability, we conducted a test–retest reliability analysis. The Spearman correlations between the first (*N* = 265) and second (*N* = 42) conducting of the Grit-HU (which happened 3 weeks apart) were identified as follows: CI: 0,628; *p* < 0.001; PE: 0,642; *p* < 0.001; Grit-HU: 0,699; *p* < 0.001. The results indicate a moderately strong positive connection between the two instances of the grit assessment with the Grit-HU. As a result, its temporal stability is reasonably acceptable.

### 4.3 The divergent validity of the Grit-HU

The divergent validity of the Grit-HU was tested by binary logistic regression, in which we investigated the unprecedented power of the cumulated grit score between national team athletes and non-national team athletes. We assume that these two groups are fundamentally different from each other on a latent dimension that assesses general performance, therefore we use it in a nominal transformation to assess the possible differentiating role of grit. The results of the analysis supported the role of grit in this differential process: the hypothetical model was significant (*χ*^2^ = 4.704; *p* < 0.05), although its explained variance was relatively low (McFadden *R*^2^ = 0.027; Cox-Snell R2 = 0.031; Nagelkerke *R*^2^ = 0.045). Grit emerged as a significant predictor with a prominent effect size (OR = 2,524; *p* < 0.05). Based on Campbell and Fiske (1959), the divergent validity of the Grit-HUwas supported.

The Mann–Whitney *U* test was also performed in order to measure the divergent validity of the Grit-HU. In the case of the factors, none of them showed significant differences between the groups of national team athletes and non-national team athletes (CI → U = 1831.500; *p* = 0.100; rrb = 0.175; PE → U = 1888.000; *p* = 0.159; rrb = 0.150), although the total score of Grit HU proved to be significant (Grit HU → U = 1681.000; *p* < 0.05; rrb = 0.236), where national team athletes (*M* = 4,155; SD = 0,419) showcased a higher level of grit than non-national team athletes (*M* = 3,975; SD = 0,476) with a medium-weak level of effect. Based on Mann and Whitney (1947) and Campbell and Fiske (1959), the divergent validity of the Grit-HU was supported again.

### 4.4 The convergent validity of the Grit-HU

The convergent validity of Grit-HU was tested with (1) correlational analysis and (2) linear regression analysis along the factors of TCI-55. The correlational analysis showed significant connections between Grit-HU, (1) Persistence, and (2) Self-Directedness. In the case of the composite score of grit, persistence showed a medium-high correlation, while Self-Directedness had a medium-low connection. While Consistency of Interest displayed a medium-low correlational relationship with Persistence and Self-Directedness, Perseverance of Effort showed a medium-high connection with persistence and a medium-low with Self-Directedness (see Supplementary Appendix Table A2).

The linear regression analysis revealed that the most considerable predictive power of Persistence and Self-Directedness appeared in the case of Perseverance of Effort and the lowest in the investigation of Consistency of Interest. The standardized coefficients were significant; their effect size ranged from weak to medium (see Supplementary Appendix Table A3). These findings highlight that the convergent validity of the Grit-HU is supported, based on Campbell and Fiske (1959).

### 4.5 The criterion validity of the Grit-HU

The criterion validity of Grit-HU was tested by correlational analysis with the use of personal evaluations about the participants provided by two external experts in the field. The experts used a five-point Likert-type scale (where the higher scores indicated the perceived performance) to express their opinions about the participants individually. We used the mean of the scores given by the experts, where no significant correlations were found neither in the case of Grit-HU (rs = 0.101; *p* = 0.638); nor regarding Perseverance of Effort (rs = 0.353; *p* = 0.083) or Consistency of Interest (rs = 0.001; *p* = 0.991). Based on Corder and Foreman (2014) and these results, the evaluation by the experts did not support the criterion validity of the Grit-HU and its subscales. In the Discussion section, we provide further interpretation for these findings.

### 4.6 Additional results

**Figure 3:**
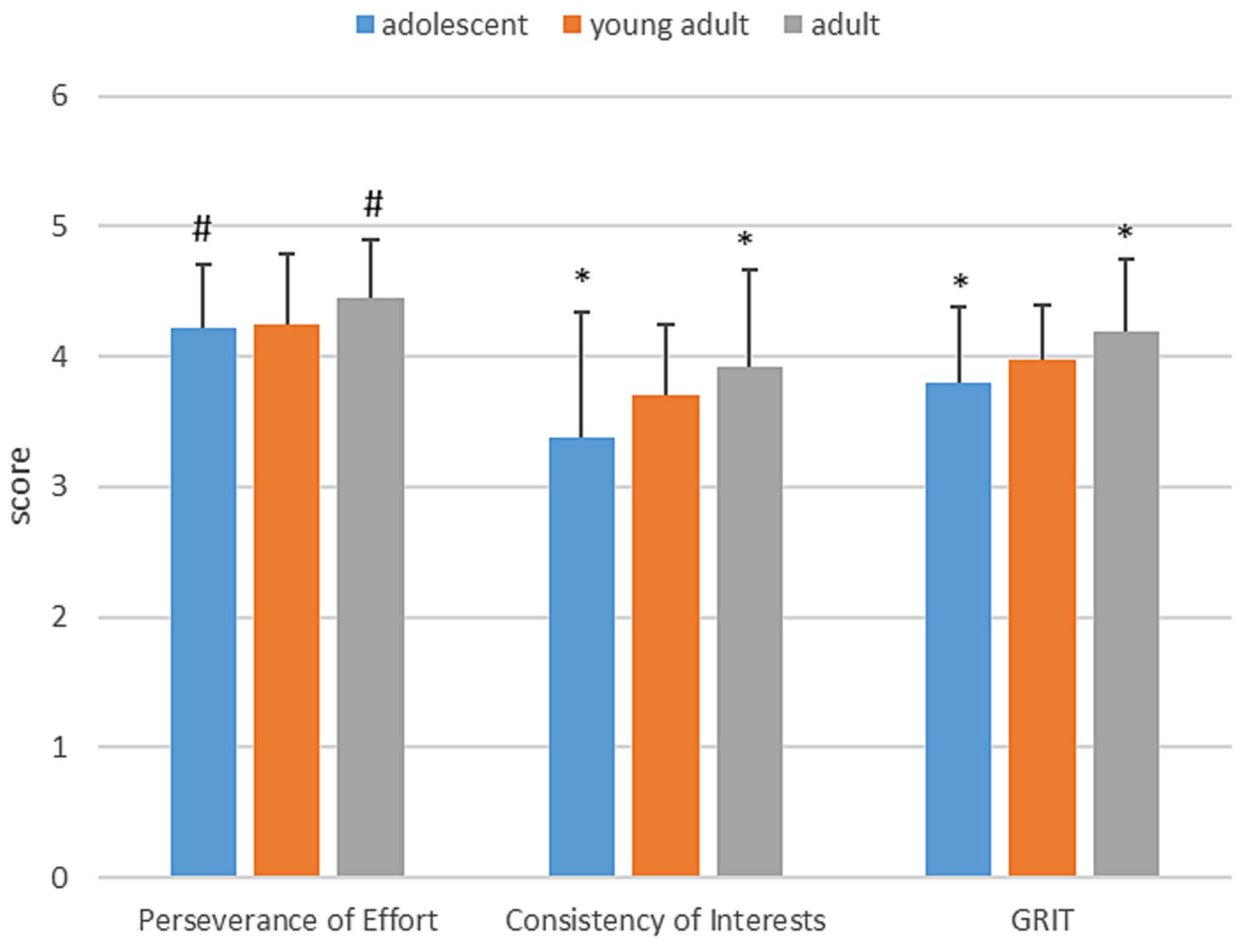
Evolution of average values and standard deviations by age group. * means a significant difference between adolescent and adult, where *p* < 0.05; # means a marginally significant difference between adolescent and adult, where 0.1 > *p* > 0.05.

In this research we also aimed to investigate grittiness according to age groups, for which the age groups were formed from the point of view of developmental psychology. The difference between the age groups (adolescent: 12–18 years; young adult: 19–25 years; adult: 26+) was tested using the Kruskal-Wallis test, which is as follows:

- There was a significant difference between the CI factor scores of the adolescent and adult categories *χ*^2^(2) = 8.537 *p* = 0.014 and the average grit scores: *χ*^2^(2) = 12.163 *p* = 0.002. In the case of the scores achieved on the PE factor, there was a marginally significant difference between these two age groups: *χ*^2^(2) = 5.633 *p* = 0.060.
- Two other age group comparisons (adolescent vs. young adult; adult vs. adult) did not show statistically verifiable differences either in the case of PE-, CI-, or the total grit score. However, an increase in average scores can be observed (see Supplementary Appendix Table A4) in the increase according to age groups (see Figure 3).

## 5 Discussion

The structure of the Grit-HU - two correlated dimensions - showed a good fit with the dimensions of the original English-language Grit Scale (Duckworth et al., 2007) after removing two items (the fourth item: “Setbacks do not discourage me.” and the seventh item: “I often set a goal but later choose to pursue a different one”). As can be seen, during the confirmatory factor analysis (CFA) of the Grit-HU questionnaire, other items showed no significant loading to any of the factors than the items of the Short Grit Scale (Duckworth and Quinn, 2009). The 2 items mentioned earlier can be found in the Short Grit Scale. That is why it is also appropriate that we used the original 12-item grit scale, so there was a greater opportunity to examine the characteristics of the sample of Hungarian athletes when measuring the characteristics of grit.

However, an interesting result is that in Birr et al’s. (2023) work, the dropped out item is the same as one of the items that did not show a significant relationship in our case (“Setbacks do not discourage me”). When they measured the Grit-S questionnaire on a sample of Portuguese athletes. This was explained by the fact that this item focuses more on grit than on one of the components of grit, persistence. We would add to this that this item also contains a kind of emotional component, which is equally true and appears in athletes as well - especially in case of losing a major match or ranking - and they experience failure or loss in the same way. Only with a solution-oriented, positive attitude, struggle, will, perseverance, passion, and goal orientation can they overcome these more easily and continue on their way to achieving their goals (this could be the reason why the item does not load well). At the same time, cultural differences and views should not be ignored, which can also explain why certain items work well in one nation and why they do not. Its internal reliability also proved to be acceptable. Therefore, in the Hungarian version of Grit Scale (GRIT-HU), we found 10 items that are related to the measured variables in accordance with the original factor structure, so only these were taken into account to calculate the total score, but in anticipation of further research, we were left with all 12 items in the adaptation of the test (Table 1).

**Table 1:** Fit indices of the proposed models.

	*χ* ^2^	df	CMIN/df	CFI	TLI	NFI	RMSEA	GFI	MFI	AIC	BIC
M1	63.461	33	1.923	0.949	0.931	0.902	0.059	0.954	0.943	6587.107	6665.526
M2	52.949	31	1.708	0.964	0.947	0.918	0.052	0.962	0.959	6580.594	6666.143

*χ*^2^, Chi-square value; df, degrees of freedom; CMIN/df, the quotient of the Chi-square value and the degrees of freedom; CFI, comparative fit index; TLI, Tucker-Lewis index; NFI, normed fit index; RMSEA, root mean square error of approximation; GFI, goodness of fit index; MFI, McDonald fit index; AIC, Akaike information criterion; BIC, Bayes information criterion.

The Grit-HU Questionnaire dimensions were proven for persistence (P) and self-directing (S), based on the fitting directional and strong correlations of the TCI-RH dimensions. Thus, Hypothesis 1 is also supported, as not only is the fit reliable but also the validation of the measured Grit dimensions with TCI-RH proves that perseverance of effort (PE) and consistency of interest (CI) are indeed measures of perseverance and self-direction. This is hardly a surprising result, partly because the aforementioned earlier studies have already proven that athletes are persistent and steady; striving with diligence and unwavering commitment in pursuit of their self-defined aspirations (Duckworth et al., 2007; Kelly et al., 2014; Larkin et al., 2015) (Table 2).

**Table 2:** Correlation matrix of Grit-HU and TCI.

	1	2	3	4	5	6	7	8	9
1. Grit-HU	—								
2. Consistency of interest	0.870^***^	—							
3. Perseverance of effort	0.838^***^	0.460^***^	—						
4. Harm avoidance	−0.040	0.014	−0.100	—					
5. Novelty seeking	−0.103	−0.117	−0.069	−0.085	—				
5. Reward dependence	0.096	0.090	0.069	0.093	0.141	—			
6. Persistence	0.523^***^	0.302^**^	0.577^***^	−0.238^*^	−0.191^*^	−0.012	—		
7. Self-directedness	0.308^**^	0.294^**^	0.298^**^	−0.190^*^	−0.046	−0.102	0.205^*^	—	
8. Cooperativeness	0.076	0.090	0.160	0.013	−0.070	0.133	0.025	0.263^*^	—
9. Self-transcendence	0.088	−0.039	0.185	−0.145	0.206^*^	0.217^*^	0.125	−0.249^*^	−0.025

*p* < 0.05; *p* < 0.01; *p* < 0.001.

In addition, persistence is one of the essential cornerstones of grit, besides determination with passion and long-term goals. Self-control (S) has previously associated with grit as a component of conscientiousness (Duckworth et al., 2007). Similar to the character scale, high achievers are characterized by reliability, trustworthiness, responsibility, and awareness. Such individuals tend to adjust their lives to their goals, giving them meaning and direction. They are long-term thinkers, who demonstrate efficacy and treat emerging situations as challenges while aspiring to make the most of themselves (Mirnics, 2006). These enumerations are very close to the description of the grit trait, thus confirming the significant association between them (Table 3).

**Table 3:** Linear regression connections among Grit-HU (and its factors), perseverance and self-directedness.

	*R*	*R* ^2^	aR^2^	AIC	BIC	RMSE	*F* (df1; df2)	*p*	*P*	P_p_	S	S_p_
Grit	0.559	0.312	0.299	086.330	097.168	0.344	34.503 (2,108)	<0,001	0.478	<0.001	0.202	<0.050
CI	0.385	0.148	0.132	166.097	176.971	0.490	09.471 (2,109)	<0,001	0.253	<0.010	0.243	<0,010
PE	0.603	0.363	0.352	102.791	113.665	0.369	31.101 (2,109)	<0,001	0.382	<0.001	0.166	<0.050

*R*, explained variance; *R*^2^, square of explained variance; aR^2^, adjusted square of explained variance; AIC, akaike information criterion; BIC, Bayes information criterion; RMSE, root mean square error; Grit, Grit Hu; CI, consistency of interest; PE, perseverance of effort; *P*, persistence; *S*, self-directedness; values under “*P*” and “*S*” are standardized regression coefficients, “*P*_p_” and “*S*_p_” are the consecutive *p*-values.

### 5.1 Coaches’ athlete assessment and grit by age groups

There was no statistically verifiable relationship between the talent map based coach’s evaluation of the Basketball Academy and the athletes’ self-reported grit results. Thus, the examined hypothesis 2 emerged as unproven in our research. This may have been caused by several factors. Coaches’ evaluations, as described in section 2.3, are grounded in the time of joint work between coach and athlete - whether it be a shorter or longer period - during which the coach gets to know his athlete. During this period, performance may fluctuate, therefore it is generally recognized that the assessment of a season cannot be considered reliable in itself, as many external and internal factors can affect the performance of average people and athletes as well. For example, a sudden injury, illness, or in some instances even a slower adaptation process might occur, if the player is away from home court, is being transferred (new environment, new hall), or if they are adapting to a new playing position. The coach’s evaluation also raises the issue of subjectivity. In the current study, for each athlete (*N* = 25), we asked two specialist coaches whose rating was 99% consistent. On the other hand, we cannot ignore the fact that we had no insight into how the evaluation was conducted, whether there was an event level or personal comparison (e.g., performance in a major competition or a more skilled teammate) when categorizing athletes. We could not find the connection between grit and the talent map that should show the possible potential of the players. As the talent map is a dynamic tool with regular revision, probably it would be more accurate to use it in longitudinal studies that span several seasons, linked with the appearance and changes of grit. Furthermore, we should not overlook the fact that the coach-athlete relationship might distort the judgment even of the most objective individual. In self-reporting tests, there is always potential for bias and it is more likely that the extent of the ability for insight and self-reflection is reduced.

We consider it a significant result that, when comparing the age groups, we obtained a significant result between adolescents (12–18 years) and adults (26+ years) regarding both the subscales (PE and CI) and the grit total score. From this we cannot yet conclude that grit scores increase with advancing age, however, since there was a statistically verifiable difference between the two extreme groups, we can say that the development of underlying skills and abilities with advancing age has an impact on the level of grit scores (Peña and Duckworth, 2018). During the sensitive period of 12–18 years, individuals undergo dramatic cognitive and physical changes while forming ever-evolving social expectations. For that reason, they paint a more realistic picture of themselves in late adolescence (17–18 years), utilizing the fundamental skills, experiences, and boundaries they have obtained (Robins and Trzesniewski, 2005; Turner and Reynolds, 2012; Duckworth, 2016; Peña and Duckworth, 2018). Likewise, the realization of the future self (Robins and Trzesniewski, 2005; Turner and Reynolds, 2012) helps to formulate and set more refined goals (ideas, philosophies, ideologies), which guide our lives and also play an essential role in the development of the grit trait (Duckworth, 2016). Taking this into account, it becomes evident that there is a tendency for higher grit scores as age progresses - as Duckworth et al. (2007), cited by Peña and Duckworth (2018) and Credé et al. (2017), cited by Peña and Duckworth (2018) have already verified between grit scores and age in adulthood in cross-sectional studies. In our study, adults (26+ years) tend to score higher than young adults (19–25 years), while adolescents (12–18 years) typically achieve the lowest points among these age groups. As Birr et al. (2023, p. 10) say, “In fact, with young athletes, considering their psychosocial development, athletic identity must be coordinated with other identities, adaptability is key and profiles with high perseverance and low consistency may occur” (Table 4).

**Table 4:** Average scores with standard deviation values by age group.

		Perseverance of effort		Consistency of interests		GRIT	
	*N*	Mean	Std. deviation	Mean	Std. deviation	Mean	Std. deviation
Adolescent	174	4.2175	0.49270	3.3753	0.96451	3.7958	0.58044
Young adult	72	4.2444	0.54460	3.70000	0.54021	3.9722	0.42167
Adult	22	4.4545	0.44156	3.9273	0.73691	4.1909	0.55713

### 5.2 National team

We investigated the relationship between national team membership and grit among the selected athletes, to confirm the focus of Sellei’s research in 2017. Consequently, particular emphasis was placed on the selection of athletes within the sampling process. We surveyed 150 athletes to inquire about their past and current selection status and 40 respondents confirmed being selected at some point. This non-proportional distribution also showed that national team member athletes score higher on the grit average than non-national team member athletes. After concluding the survey, we examined whether grit can be utilized to predict the likelihood of an individual becoming a national team member. In the Results section, we can see that this indicator is 4.5%, aligning closely with the 4% measured by Duckworth et al. (2007), confirming our hypothesis 3.

## 6 Conclusion

The objective of the present study encompassed the analysis of the Grit-HU Questionnaire on an athlete population, comparing the grit trait with the coach’s evaluation, in addition to assessing the predictive power of grit in terms of an athlete’s likelihood of being selected for the national team. The obtained results suggest that, according to the psychometric indicators, the questionnaire demonstrates adequate validity and reliability. In our study, we used a multilevel approach: the first model (M1) includes only the direct effects on the items for the first-order factors, and then the effects of CI and PE on the particles. In the second model (M2) we used three residual covariance correlations between the items. M2 yielded satisfactory fit indices, therefore we accepted it as our final model which is why we chose this as our final model. This model confirms the assumption that the grit trait is a multifactorial concept. From a practical point of view, if we want to strengthen or develop grit in an individual, we need to grasp the components that have a direction through interests; and the amount of energy invested by effort. What both components have in common: focus and organization. These skills can be part of the skill palette of the grit trait.

Secondly, the self-reporting Grit-HU Questionnaire cannot be compared with the coach’s evaluation it is based on other criteria. Lastly, national team athletes achieve a higher average score on the measured Grit dimensions than their non-national team counterparts, which solidifies the predictive power of grit at 4.5% for national team qualification, as compared to the 4% measured by Duckworth et al. (2007). As a result, the phenomena examined in this research further expand the knowledge about the personality trait of grit, thereby enriching the colorful palette of the positive returns it yields. Researchers are trying to reinvent the Grit Scale with several models (e.g.: GSCA, Academic Grit Scale, PE-Grit), often by adapting to the specifics of their research field. Clark and Malecki (2019) created the Academic Grit Scale specialized to the field of pedagogy; Schimschal et al. (2022) designed the Grit Psychological Resources Scale in the nursing environment; Zhang et al. (2023) developed the Physical Education Grit Scale, in which model they separate academic “grit” from athletic “grit,” trying to distinguish between the grit that appears in different fields. These attempts point in the direction that each research field would require a specific version of the grit scale.

However, along the lines of these efforts, we deviate from the fact that grit is field-specific, grit generally helps the individual to achieve outstanding results, and the effect of grit can appear, e.g., in music, and school performance (Duckworth et al., 2007). Our current research also tries to point out that the original 12-item Grit Scale, similar to Shaban (2020), shows a suitable fit, and produces adequate results in terms of measurement, so it can be applied to the sample of athletes as well, from adolescence onwards. Keeping this model and factor structure, we would like to contribute to the possibility of an international comparison of the Grit Scale. Referring to the model fit statistics described by Birr et al. (2023), regarding the future of research and the Grit-HU Questionnaire, as further direction we consider measurements with the Short Grit Scale on a sample of Hungarian athletes.

Given that grit appears to be very useful for coaches when making decisions about talent identification and development in athletes (Tedesqui and Young, 2020) and also that grit is a trait that can be developed (Bashant, 2014; Duckworth, 2016), our study can provide a distinctive utility to researchers and practitioners. Grit-HU can provide important help in identifying features of the athletes that could affect their involvement in sports. Measuring grit can be a potential contribution to raising the attention of coaches about the impact of these variables on the behavior of the athletes helping them to promote passion and consistency. Research findings revealed that grit is a statistically significant predictor of subjective well-being (Akbag and Ümmet, 2017). Overall, focusing on grit is not only important from the point of view of sports success but can be crucial for the mental health of athletes. In addition, it should be noted that the sports environment is a very complex area (Birr et al., 2023). Almost every sport has various characteristics (e.g., from what age can you start, when is the retirement age, what is the competition system like, etc.) these can influence the development of personality and the development of specific abilities as well (Cormier et al., 2021; Birr et al., 2023), therefore it is essential to start longitudinal studies to measure the development of grit personality trait in an athlete population.

Future directions include the amelioration of the Grit-HU for athletes, as well as the implementation of novel interventions by coaches and sport psychologists that may improve athlete grit. We also recommend that research and practical colleagues in the field of sports psychology start research on grit in the future, since the preservation and protection of the athletes’ physical and mental health not only affect the coaching duties, but are also part of the sports psychology work.

## 7 Limitations

First of all, the sample size is large, close to 300 people, but for an even more accurate measurement, it will be necessary to expand the number of employees in the future and include even more athletes - in both individual and team sports, as well as national team members. The sample of elite and young athletes included many different sports. While this heterogeneity and wide immersion support generalizations and validation in the sports context, it may also mask possible differences across sports. Future studies could examine possible differences in the predictive value of grit across sports domains. Furthermore, the number of athletes who were evaluated by 2 coaches is very low. It is important to increase this number of elements to approximately 100 people and conduct the evaluation for at least 2 seasons to observe the changes. Thirdly, considering the future of research, it would be worthwhile to include the examination of the coach-athlete relationship. These duos have a lot of power (Jowett and Ntoumanis, 2004; Kovács et al., 2021) that can either benefit or hinder the detection of grit. The work between coach and athlete includes getting to know each other, being attuned to each other, working, and fitting in. These factors can greatly influence the extent to which an athlete can show their innermost core of personality, which includes grit (comp.: manifested vs. latent), and the extent to which a coach can see this even when it is not yet manifested.

## Data availability statement

The original contributions presented in the study are included in the article/Supplementary material, further inquiries can be directed to the corresponding author/s.

## Ethics statement

The studies involving humans were approved by the PTE BTK-Egyesített Pszichológiai Kutatásetikai Bizottság (United Committee on Psychological Research Ethics of University of Pécs). The studies were conducted in accordance with the local legislation and institutional requirements. Written informed consent for participation in this study was provided by the participants’ legal guardians/next of kin.

## Author contributions

AA: Funding acquisition, Investigation, Project administration, Resources, Writing – review & editing. NF: Conceptualization, Formal analysis, Investigation, Methodology, Resources, Writing – original draft. SB: Funding acquisition, Data curation, Formal analysis, Software, Validation, Visualization, Writing – review & editing. RJ: Conceptualization, Formal analysis, Investigation, Methodology, Resources, Supervision, Writing – original draft.

## Footnotes

^1^GPA is a system used in high schools and universities, mainly in the US, to measure a student’s performance and academic achievement. GPA is calculated by converting grades or percentages you receive for an assignment (A, B, C etc) to a corresponding point on the GPA scale. Most high schools and universities report grades on a 4.0 scale. The top grade A, equals a 4.0 (McCabe, 2023; https://www.timeshighereducation.com/student/advice/what-gpa).

^2^SAT means *Scholastic Aptitude Test* or *Standard Assessment Task* and is widely used in college admissions in the United States, developed by the College Board. It measures mathematical and verbal reasoning (Wikipedia, 2024; https://en.wikipedia.org/wiki/SAT).

^3^ACT means American College Testing is a standardized test used for college admissions in the United States. It measures 4 academic skill areas: English, mathematics, reading, and scientific reasoning [Wikipedia, 2024; https://en.wikipedia.org/wiki/ACT_(test)].

^4^ https://sites.sas.upenn.edu/duckworth/pages/research

^5^ https://www.jamovi.org
