# Supplementary material for: Investigating the effect of grit trait on performance and success in Hungarian athlete’s sample
Source: Front Psychol. 2024 Apr 12;15:1283115. doi: 10.3389/fpsyg.2024.1283115 (PMC11048980; doi:10.3389/fpsyg.2024.1283115)
Supplement: Supplementary file 1 [file Table_1.pdf]

Appendix A

GRIT questionnaire translated into the Hungarian language.

Kérjük, válaszoljon a következő 12 mondattal kapcsolatban, hogy mennyire tartja magát olyannak! Legyen őszinte, nincsenek sem jó, sem rossz válaszok!

	**Egyáltalán nem vagyok ilyen**	**Nem vagyok ilyen**	**Egy kicsit ilyen vagyok**	**Inkább ilyen vagyok**	**Teljes mértékben ilyen vagyok**
1, Addig próbálkozom valamivel, amíg meg nem csinálom.	1	2	3	4	5
2, Belekezdek egy új dologba úgy, hogy a többit nem fejezem be.	1	2	3	4	5
3, Az érdeklődésem havonta változik.	1	2	3	4	5
4, Nem leszek rosszkedvű, ha valamit nehéz megcsinálni.	1	2	3	4	5
5, Volt olyan dolog, amit nagyon szerettem csinálni, de már nem érdekel.	1	2	3	4	5
6, Kitartó vagyok.	1	2	3	4	5
7, Gyakran tűzök ki célokat, de később váltani tudok egy újra.	1	2	3	4	5
8, Nem érdekelnek az olyan dolgok, amit sokáig tart befejezni.	1	2	3	4	5

9, Bármit befejezek, amit elkezdtem.	1	2	3	4	5
10, Be tudtam fejezni olyan dolgot, amit sokáig kellett csinálni.	1	2	3	4	5
11, Szinte havonta találok magamnak új elfoglaltságokat.	1	2	3	4	5
12, Szorgalmas vagyok.	1	2	3	4	5
