# Supplementary material for: Investigating the effect of grit trait on performance and success in Hungarian athlete’s sample
Source: Front Psychol. 2024 Apr 12;15:1283115. doi: 10.3389/fpsyg.2024.1283115 (PMC11048980; doi:10.3389/fpsyg.2024.1283115)
Supplement: Supplementary file 2 [file Table_2.pdf]

Appendix B

TCI-55 questionnaire in Hungarian language

Ebben a kérdőívben olyan állításokat talál, amelyeket az emberek gyakran használnak saját véleményük, hangulatuk, viselkedésük vagy lelki állapotuk jellemzésére.

Ez a kérdőív nem „vizsga”: nincsenek benne helyes vagy helytelen, „jó” vagy „rossz” válaszok, hiszen az emberek érzései és véleményei különbözőek. Ezért habozás nélkül tegyen X-et minden esetben az Ön személyéhez közelebb álló válaszokat, és ne legyen tekintettel arra, hogy mit gondolnának ugyanarról mások.

Kérjük, a következők alapján próbálja meg eldönteni, hogy az adott állítás mennyire illik Önre:

1- igaz,

2- nem igaz.

**Sorszám**	**Állítás**	**igaz**	**nem igaz**
1	Mindig bízom a dolgok kedvező kimenetelében, még olyankor is, amikor a legtöbben aggodalmaskodnak.		
2	Élvezettel állok bosszút azokon, akik megsértenek.		
3	Sokszor úgy érzem, hogy az életemnek nincs különösebb célja vagy értelme.		
4	Általában inkább a magam útját követem, mintsem a mások kívánságait teljesíteném.		
5	Nem túlságosan kedvelem azokat, akik másképpen gondolkodnak, mint én.		
6	Érzéseimet, élményeimet jobban szeretem nyíltan megbeszélni a barátaimmal, mint magamban tartani.		
7	Mások érzéseit ugyanannyira figyelembe szoktam venni, mint a sajátjaimat.		
8	Sokkal jobban szeretem a „régi, jól bevált” módszereket, mint az „új, modern” eljárásokkal való kísérletezést.		
9	Gyakran azt kívánom, bárcsak én lennék a legokosabb a világon.		
10	Élvezem, ha az ellenségeimet szenvedni látom.		
11	Rendszerint másoktól várom, hogy ők oldják meg az én problémáimat.		

12	Nem érdekel különösképpen, hogy mások kedvelnek-e engem vagy a módszereimet.	
13	Az emberek többségét képtelen vagyok igazából megérteni.	
14	Idegenek társaságában félénkebb, gátlásosabb vagyok másoknál.	
15	Szentimentálisabb vagyok, mint a legtöbb ember.	
16	Döntések előtt szeretem hosszasan végiggondolni a dolgokat.	
17	A legtöbb embernél keményebben dolgozom.	
18	Szívesen állok mások rendelkezésére.	
19	A problémáimat legszívesebben megtartom magamnak.	
20	Azoknak sem tudok nyugodt szívvel ártani, akik pedig velem szemben tisztességtelenek voltak.	
21	Gyakran azt kívánom, bárcsak örökre fiatal maradhatnék.	
22	Időnként úgy érzem, mintha része lennék egy térben és időben végtelen valaminek.	
23	Néha úgy érzem, hogy szavakkal megmagyarázhatatlan, közvetlen lelki kapcsolatba kerülök másokkal.	
24	Mielőtt döntenék, általában minden körülményt aprólékosan végig szoktam gondolni.	
25	Sokszor úgy érzem, mintha egészen eggyé válnék a körülöttem lévő világgal.	
26	Mások engem túlságosan irányítanak és befolyásolnak.	
27	Legtöbbször a túlzásokat, sőt a valótlanságot is el tudom hitetni az emberekkel.	
28	Nagyon megindítanak az érzelmekre (pl. a szegények vagy a sérült gyermekek megsegítésére) irányuló felhívások.	

29	Mindig jobban hajtom magam másoknál, mert mindent a lehető legjobban akarok megcsinálni.	
30	Gyakran azért nem tudok a bajokkal megbirkózni, mert egyszerűen nem tudom, mit tegyek.	
31	Sokkal jobban szeretem költeni, mint félretenni a pénzt.	
32	Ügyesen tudok lódítani egy kicsit, ha valakit meg akarok tréfálni, vagy ha egy történetet hatásosabban akarok elmesélni.	
33	Változó körülményekhez nagyon nehezen tudok alkalmazkodni, mert olyankor feszült, ideges és fáradékony leszek.	
34	Nehezemre esik elviselni a tőlem különböző embereket.	
35	Nem hiszem, hogy az életemnek különösebb célja vagy értelme volna.	
36	Mindig igyekszem tőlem telhetően együttműködni másokkal.	
37	Szokatlan helyzetben akkor is feszült és ideges szoktam lenni, ha mások szerint semmiféle veszély nem fenyeget.	
38	Gyakran túlságosan is önállónak tartanak, mert nem azt teszem, amit mások szeretnének.	
39	Szinte minden társaságban magabiztos, határozott szoktam lenni.	
40	A barátaim sem ismerik igazán az érzéseimet, mert csak nagyon ritkán mondom el a személyes véleményemet.	
41	Nem vagyok hajlandó mások kedvéért a magam útjáról letérni.	
42	Számos rossz szokásom nehezíti meg fontos és értékes céljaim elérését.	
43	A tetteimet többnyire rajtam kívül álló tényezők határozzák meg.	
44	Szomorú filmeken könnyebben sírok, mint a legtöbb ember.	
45	Még sokat kell gyakorolnom, amíg kifejlődnek bennem a pillanatnyi kísértéseknek biztosan ellenálló helyes szokások.	

46	Mindenkihez tisztelettel és méltósággal kell közeledni, még ha jelentéktelennek vagy hitványnak látszik is.	
47	Nekem többnyire sikerülni szokott, amibe belefogok.	
48	Szeretek új dolgokat, új módszereket kipróbálni.	
49	Jobban szeretek takarékoskodni, mint szórakozásra, mulatságra elkölteni a pénzt.	
50	Személyesen is éreztem már kapcsolatot titokzatos természetfeletti, isteni erővel.	
51	Voltak olyan boldog pillanataim, amikor világosan és tisztán egynek éreztem magam minden létezővel.	
52	Sokszor úgy érzem, mintha része volnék egy hatalmas és minden életet irányító szellemi erőnek.	
53	Még barátok között sem szeretem túlságosan kiadni magam.	
54	Többnyire feszült és ideges leszek, ha valami új és ismeretlen dologba kell fognom.	
55	Még egy kisebb betegség vagy stressz után is több az energiám és az önbizalmam, mint a legtöbb embernek.	
